# Adhoc mobile power connectivity using a wireless power transmission grid

**DOI:** 10.1038/s41598-021-97528-5

**Published:** 2021-09-09

**Authors:** Pawan Gaire, Dieff Vital, Md Rayhan Khan, Cherif Chibane, Shubhendu Bhardwaj

**Affiliations:** 1 Department of Electrical and Computer Engineering, Florida International University, Miami, 33174 USA; 2 WiGL inc., Hampton, VA 23666 USA

**Keywords:** Engineering, Electrical and electronic engineering

## Abstract

Wireless charging of devices has significant outcomes for mobile devices, IoT devices and wearables. Existing technologies consider using Point to Point type wireless transfer from a transmitter Tx (node that is sending Power) to a receiver Rx (node that receives power), which limits the area of coverage for devices. As a result, existing systems are forced to use near field coupling to charge such devices. Fundamental limitation is also that such methods limit charging to a small hotspot. In partnership with Wireless Electrical Grid LANs (WiGL pronounced “wiggle”), we demonstrate patented Ad-hoc mesh networking method(s) to provide wireless recharging at over 5 feet from the source, while allowing significant lateral movement of the receiver on the WiGL (Wireless Grid LAN or local area network). The transmitter network method leverages a series of panels, operating as a mesh of transmitters that can be miniaturized or hidden in walls or furniture for an ergonomic use. This disruptive technology holds the unique advantage of being able to provide recharging of moving targets similar to the cellular concept used in WiLAN, as opposed to prior wireless charging attempts, which only allow a hotspot-based charging. Specifically, we demonstrate the charging of a popular smartphone using the proposed system in the radiating near field zone of the transmitter antennas, while the user is free to move in the space on the meshed network. The averaged received power of 10 dBm is demonstrated using 1W RF-transmitter(s), operating in the 2.4 GHz ISM band. The proposed hardware consists of antennas arrays, rectennas, power management and USB 2.0 interfaces for maintaining a voltage between 4.2 and 5.3 V and smooth charging. We also show extending the wireless grid coverage with the use of multiple transmitting antennas, and mechanical beam-steering even further an increased coverage using the proposed system.

## Introduction

Microwave radio frequency (RF) technology has provided transformative changes in our society via innovations such as wireless communication, radio wave sensing and wireless power transfer^1–4^. Specifically, for power needs of mobile devices, RF technology has offered a new vision of wirelessly powered world^5^. This can be realized through a wireless power transmission grid, which could power a range of devices from traditional mobile phones to wearable health and fitness devices^6^, implantable devices^7^, and other Internet of Things (IoT) type devices^8^. This vision is specially becoming true on account of ever reducing power usage of modern electronics and innovations in rechargeable batteries. With the realization of this technology, devices may no longer need a battery (or maybe a small one), and lead way to a new generation of batteryless devices. This is important since in current mobile electronics, batteries are a significant cost driver.

**Figure 1:**
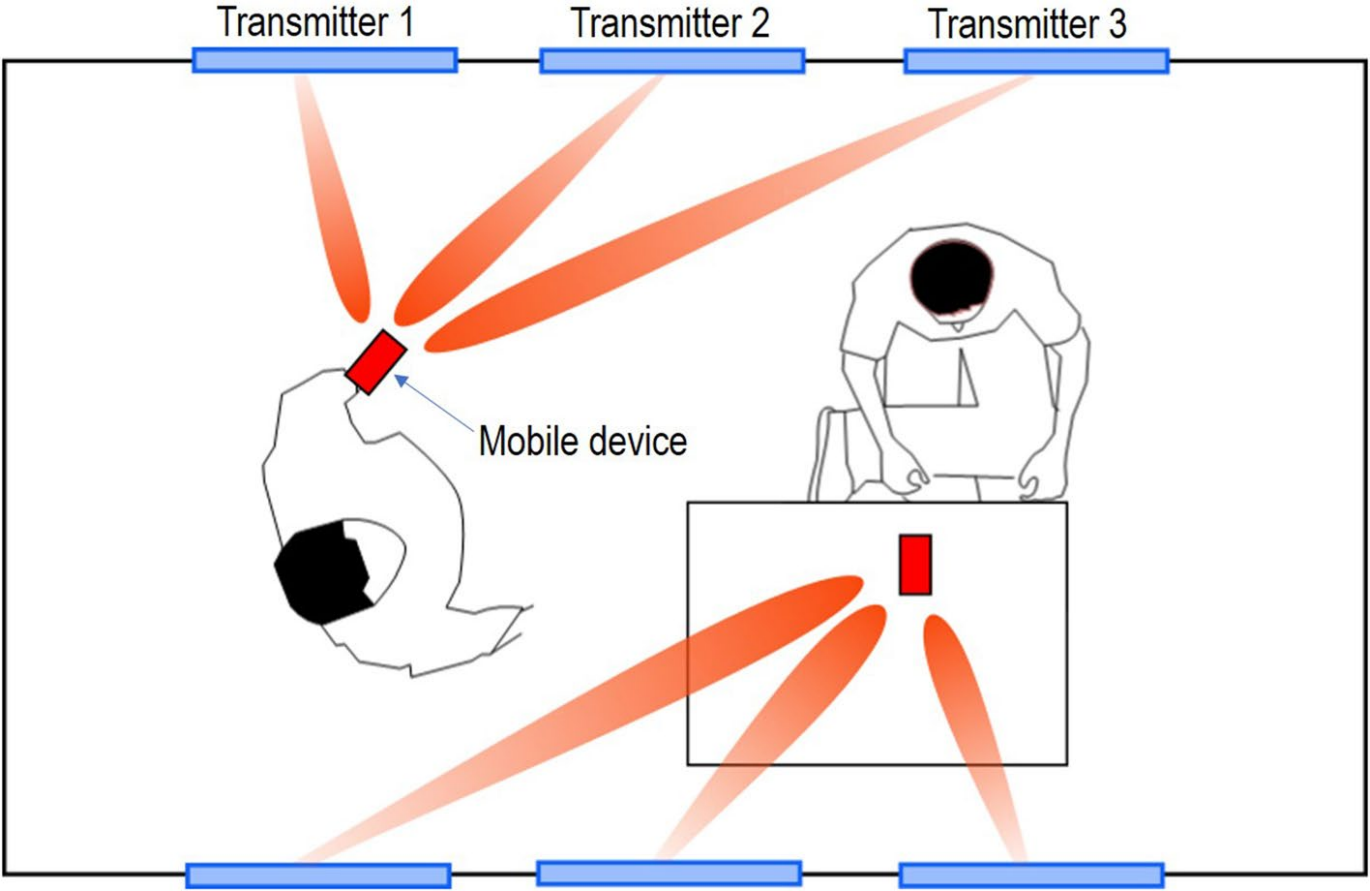
An example showing a wireless power transmission grid for home use application for continuous powering of mobile devices. Figure drawn using Inkscape 1.1 available from https://inkscape.org.

Because of the growth of mobile computing and wearables, the demand for wireless source of power is increasing for the scenarios where cable-based charging is not feasible or where the issue of battery depletion and replacement exists^9^. Among wireless approaches, magnetic near-field wireless charging is popular. But in this modality, the wireless charging distances are limited to few centimeters^10,11^. For the most ergonomic use, the wireless charging up to several feet is necessary as it will enable the charging of devices for users who are engaged in daily life activities^12,13^. The application of such a system is shown in Fig. 1 where the user is able to wirelessly charge their smartphone while moving around the room and using the device as opposed to having their phone immobile on top of charging pad. This is the application where the radiative near-zone and far zone charging methodologies are becoming mainstream while using the open ISM frequency bands and adhering to the power limits in accordance with FDA and FCC guidelines^14^. A second crucial issue is charging during significant misalignment between the source and receiver, providing a wider region of charging, as compared to specific hot-spot charging^15,16^. This is crucial step in achieving a practical wireless power grid.

In this presented work, our goal is to solve crucial wireless charging issues from a practical design standpoint, while providing sufficiently high power for mobile phone-charging and through a grid of transmitters. Comparing to past work, we distinctively proposed a system which is capable of fulfilling the higher power needs for mobile-phone charging through a multi-transmitter system. In the past, wireless powering systems have focussed on low power systems for implantable medical devices^17^, integrated circuits^18^, wearable/fitness devices^19^, and other IOT devices^8,20^. These applications generally rely on fixed distance, low-power charging, requiring significantly reduced requirements for antenna, and hardware design. Specific to our practical needs, to cater to higher power and mobile use, new transmitter receiver configurations, grid based charging concept and related investigations on distance and beamsteering are shown in this paper. Specifically, we demonstrate wireless power transfer system for delivering power to a smartphone while the user is mobile inside the area of 8 $\times$ 10 feet. Furthermore, the beam steering studies in multiple transmitter environment are shown, which reveal that in order to save propagation losses, increased directivity and gain of antenna could be combined with beam steering such that the narrow and directive beam could be steered in the direction of target. Even with wide use of beam steering concept^21–24^ for communication application, its use for RF power transfer is shown. The proposed work indeed represents a practical representation of the prior patents by the authors^25^.

Power charging requirement of commercially available phones are significant. Typically, consumer electronics smartphone battery requires power level between 20 mW and 1.3 W, which is higher than the power usage of the sub-mW sensor nodes, and other IoT devices^1^. Therefore, wireless powering solution requires well-defined and yet limited power to be transmitted efficiently to a targeted device. In presented work, we consider use of low frequency radio wave (as opposed to mm-wave^26^ which could have more path loss) and use of directive antennas arrays as an effective method for enable charging^27,28^. We further introduce mobile aspect by using many transmitters, which can provide a seamless power connectivity during significant movement of up to several feet.

Here, we demonstrate a wireless power transmitter grid or Wireless Grid local area network concept, which uses an Ad-hoc mesh to cover an area for wireless charging. The demonstrations use individual transmitter panels and provide a prototype demonstration that many transmitters can be used to cover an area for seamless charging of a commercial smartphone. Specifically, we show that the charging from three transmitter panels can provide a coverage of an area of 8 feet by 10 feet. We further also show that the beam-steering capability provides a cumulative charging from more than one transmitter and maintains continuity of charging with movement. The paper will detail the design of directive RF transmitter at 2.4 GHz, receiver antenna, rectifier and power management, and USB 2.0 charging interface design. Finally, a system integration and experimental demonstration results are presented.

**Figure 2:**
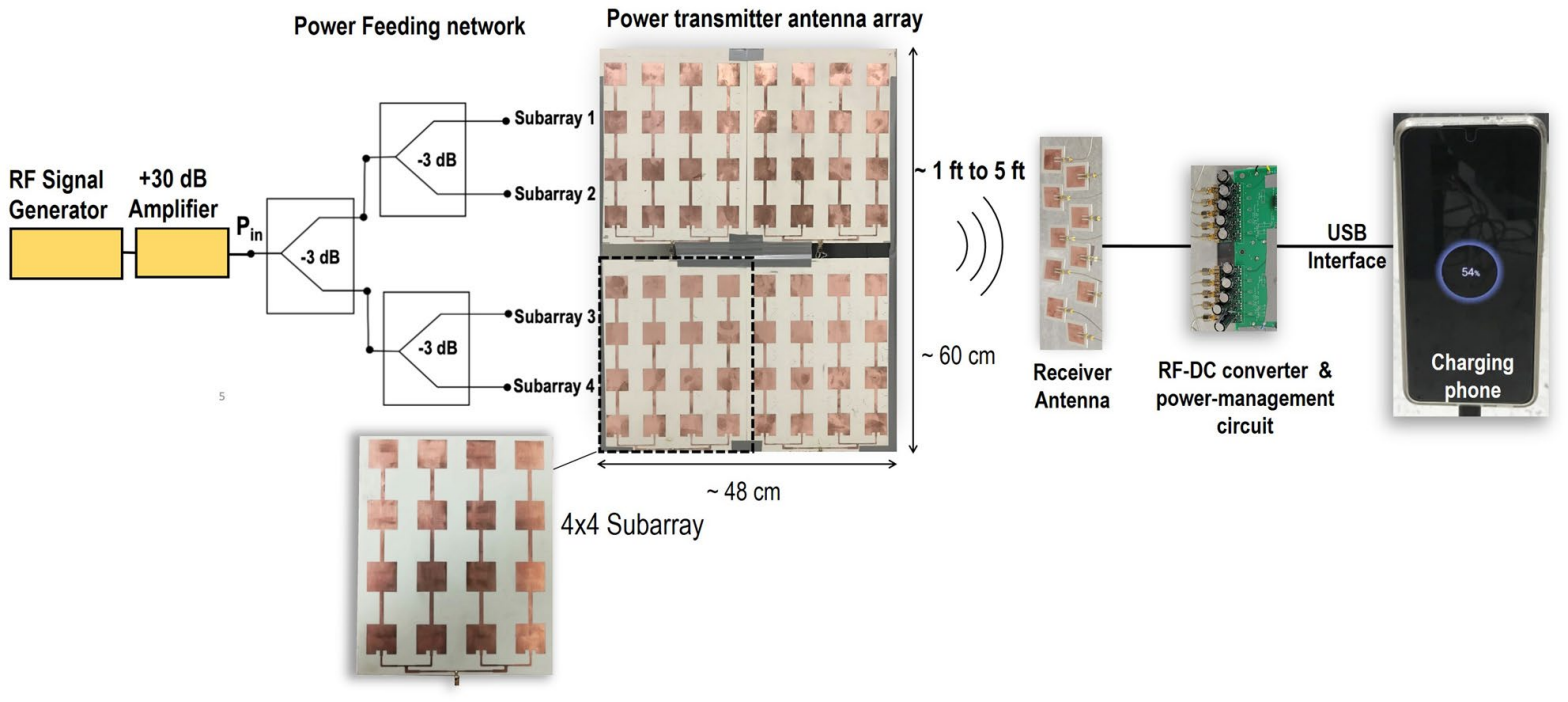
Wireless power transfer source panel design for the realization of in-home wireless grid. Figure drawn using Inkscape 1.1 available from https://inkscape.org, and integrated with real antenna, rectifier and phone photographs.

## Overview of power grid system

The proposed vision shown in Fig. 1 can be realized using individual transmission source points which can provide the required power transmission capability using the radiative near-zone and far-zone fields. We propose a system shown in Fig. 2 to realize one such source points by using a planar antenna array which is easily hidden into walls, or items of furnishing. The system consists of continuous wave source (Tx) to generate RF power at 2.4 GHz, which is amplified to approximately 1 W power (in compliance with FCC regulations^14^) and fed into an antenna array with 22 dBi gain. The RF power is wirelessly transmitted to a remote receiver which is located within several feet of the source and consists of array of rectenna elements along with power management circuits to allow for DC power combining. To enable a continuous charging which requires approximately 5 V, we have used commercial off-the-shelf (COTS) power management circuit, which uses a store-and-dump scheme to provide a 5 V DC output. Furthermore, to allow for current enhancements for efficient charging, we used parallel connection of the rectenna arrays. Therefore, crucial design blocks to enable this system are the transmitter array, design and integration of receiver array, power management circuit, and USB charging interface for efficient connection to mobile phone charging. We then characterize the system using several panels and report the results of the wireless charging under mobile conditions.

### Design of transmitter panel

To achieve wireless charging for up to several feet, while keeping the transmitted power to be within safe and FCC allowed power level of 1 W, we anticipate an antenna gain requirement of 20 dBi or more. This led us to the design of a planar array antenna containing $8\times 8$ patch antenna elements as shown in Fig. 2. This requires feeding of all 64 elements, without significant loss while providing a low-profile, compact, and cost-effective design. To achieve this, we designed four independent antenna panels consisting of antenna subarray with $4\times 4$ patch elements. The subarrays were fed through a network of power dividers enabling single feed input to feed all four panels. Each sub-array panel consisted of four $1\times 4$ serially fed patches. This allowed for avoidance of a complex corporate feed network, which could result into a multilayer design and additional cost.

**Figure 3:**
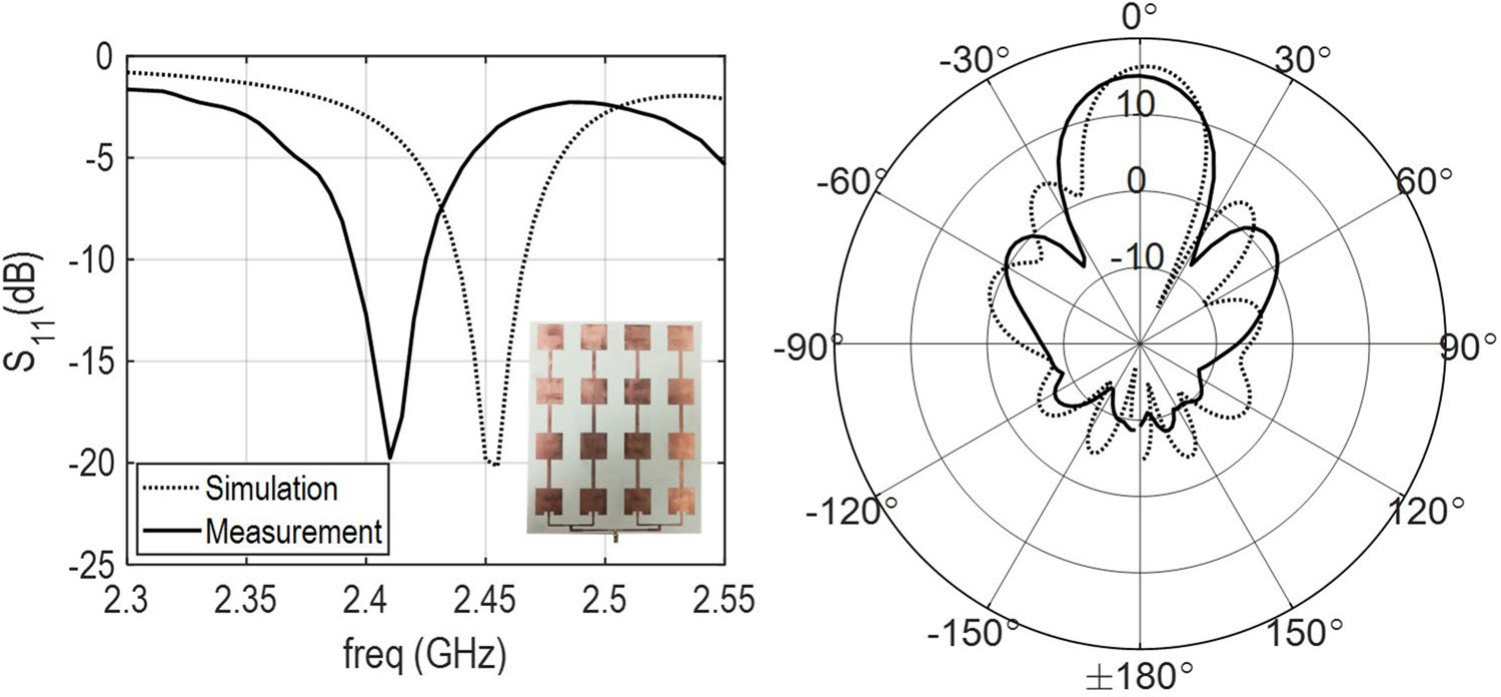
Reflection coefficient (*left*) and gain pattern (*right*) measurements for the 4$\times$4 subarray. Photograph of the array under measurement is shown in the inset. Plots generated using Matlab R2019b available at https://mathworks.com. The figure was drawn using MS-PowerPoint 2016.

The subarray design was done using full-wave electromagnetic simulations to allow for the optimization of the return loss and gain of the subarray. Low loss substrate, Rogers RO4003 ($\epsilon _r=3.38$), with commercially available thickness of 1.52 mm was used. Reflection coefficient for $4 \times 4$ subarray shows an excellent matching at 2.4 GHz as shown in Fig. 3, however we notice a shift from the design by 300 MHz, likely arising from the tolerances in the substrate modeling and fabrication tolerances in the metal etching process. The gain and pattern of the subarray is plotted in Fig. 3 and shows 16 dBi maximum gain in the broadside. This also suggests that with four such subarrays, the total gain of the transmitter array should be close 22 dBi.

### Power transmission capability of the transmitter antenna

**Figure 4:**
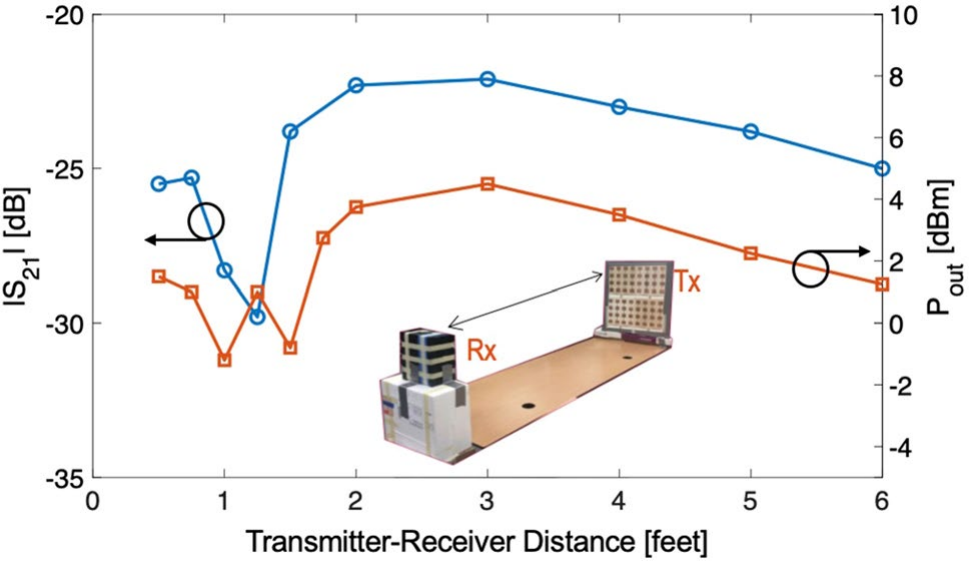
Transmission capabilities of the developed transmitter using a single element receiver as a function of distance between the Tx and Rx. Plot generated using Matlab R2019b available at https://mathworks.com.

To understand the power transmission capabilities, first we record the power transmission from the transmitter antenna as a function of distance from the receiver. For this experiment a single patch antenna with 7 dBi gain was used as receiver for preliminary tests of RF power transfer. We measure the transmission coefficient at 2.4 GHz and the receiver RF power as a function of distance. The distance between the transmitter and receiver was varied from 0.25 feet to 6 feet. The experimental setup is shown in Fig. 4. An RF power loss (S$_{21}$) raging between 22 and 30 dB was observed under this RF transmission. The received power is low also due to single antenna reception which can be improved by having multiple receiver antennas. We also note a decrease in power when the receiver is very close to transmitter since the array’s beam is not well formed in the near field and direct near field coupling results is smaller transmission.

Received power was measured under the case when the transmitted power of 30 dBm (1 W), the S$_{21}$ and received power as a function of distance is shown on Fig. 4. The received power was recorded using a spectrum analyzer. As shown on the graph, power as high as 4.5 dBm (3 mW) is obtained. For increasing the received power, a receiver antenna with larger aperture is required. Specifically, this is achieved by using multiple receiver antennas. These investigations were further pursued to obtain an efficient design.

### Efficient RF to DC converter with store and dump power management circuit

**Figure 5:**
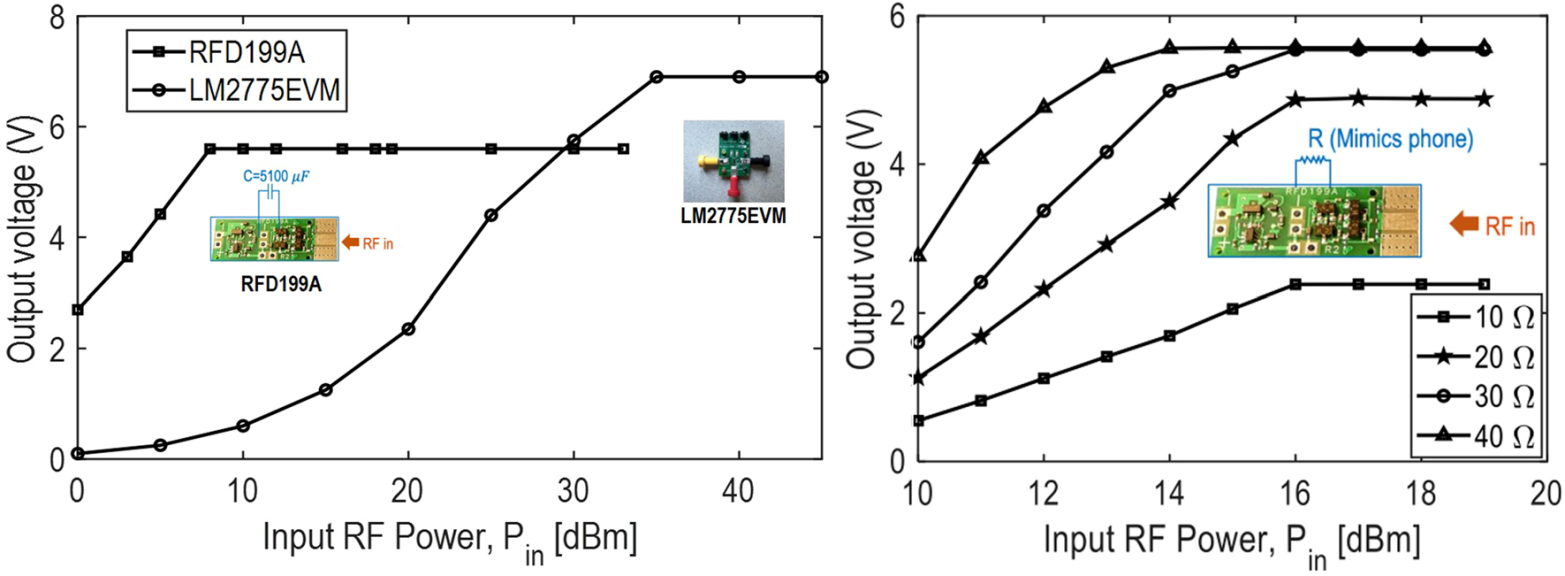
Comparison of the chosen rectifier + power management topologies for the proposed receiver system. Plot generated using Matlab R2019b available at https://mathworks.com.

Two different available rectifiers with power management units were tested to find the best fit for charging a smartphone. These units were commercially available LM2775EVM TI power management IC, and rectifier and power management module based on COTS RFD199A. The signal generator was setup to provide an input RF power signal at 2.4 GHz. True RMS multimeter which records the RMS mean voltage, was used to record the output DC voltage at the output of each module. The comparison of output DC voltage versus input RF power is shown in Fig. 5. LM2775EVM provided the required output voltage of 4.5 V for charging when input power is at least 25 dBm. Furthermore, the output voltage saturated for an input beyond 35 dBm to an output of 6.9 V. Although this module can be used to regulate the output voltage, the required input power is too high to reach the needed voltage value. On the other hand, RFD199A is capable of regulating the voltage at 5.598 V, while the input power is as low as 8 dBm. RFD199A was characterized until 33 dBm which is the maximum input power it can handle. A minimum of 6.3 mW of RF power is required to obtain 5.598 V which is required by the USB standard and is suitable to charge a phone.

RFD199A, if used with a storage capacitor, takes RF at 2.45 GHz signal and converts to pulsed DC voltage signal in the range of 5.3 V to 4.2 V. The signal is continuous for high power, while it is pulsed with a decreasing duty cycle for input RF signal with decreasing power. The module is made of two parts, namely rectifier and the power management system that is made up of a voltage-controlled switch. When the voltage across the storage capacitor reaches 5.3 V, the voltage-controlled switch turns on to dump the power into the load and turns off and when it drops below 4.2 V. The output is continuous if enough power is available such that the voltage across the capacitor does not drop below 4.2 V. Hence, the power management ensures that the voltage across the load is in the range of 4.2 V and 5.3 V which is suitable to charging the phone.

The output current from the RFD199A module is crucial for effective charging. The current drawn from the module is a function of load resistance connected to the module. Knowing that the input impedance of smartphones is $<~50~\Omega$, we analyze the output voltage under such low load conditions. This is shown in Fig. 5. As shown, the output voltages between 2 and 5 V are possible in this variation of the load using the proposed module. Although this output voltage is characterized without an output capacitor and use of storage capacitor was found to improve overall voltage level.

### Developing USB 2.0 charging interface

**Figure 6:**
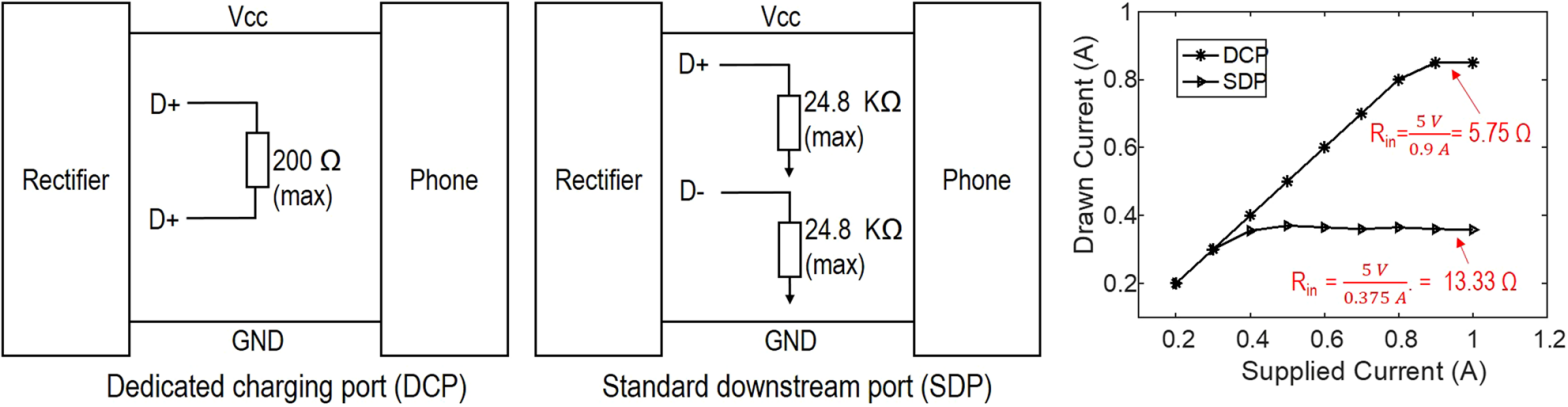
Standard charging modalities of a USB 2.0 interface, namely DCP and SDP, and comparison of measured input resistance. Plot generated using Matlab R2019b available at https://mathworks.com. The figure was drawn using MS-PowerPoint 2016.

Exact input impedance of the load (phone) depends on the USB charging interface. We study 2 different charging configuration using USB 2.0 interface connected to a smartphone. These methods are (1) dedicated charging port (DCP) configuration and (2) standard downstream port (SDP) configuration^29^. The schematic for the two configurations is shown in Fig. 6. DCP is used for charging from the power plugs, where charging takes place without digital communication. DCP is identified by shorting of the D+ and D- lines, which allows any USB cable as replacement for charging, and allows maximum current supply of 1.5 A. SDP, on the other hand, can be enabled by D+ and D− lines grounded separately through 24.8 k$\Omega$. The maximum load current, therefore, is 2.5 mA while suspended, and maximum of 500 mA when the devices is connected. Hence, these two configurations are related to low current (SDP) and high current (DCP) charging conditions. But what matters the most in our case is the input impedance that we observe in each case. Higher impedance is preferred for us for selection of rectifier design.

We setup an experiment using SDP and DCP configurations for a comparative measurement of the input resistance of the two cases. This input impedance will act as a load for the rectifier and power management circuit. For this measurement, we supply 5 V through the USB cable while varying the supplied current. Then we measure the current drawn by the smartphone. We found that, DCP allows saturation of the drawn current at 0.9 A, while SDP restricts it to 0.375 A. But I–V ohms law for saturation region shows that resistance for the DCP is smaller than SDP. The low current charging mode has input impedance of 13.33 $\Omega$, which is better for rectifier design, as rectifier efficiency increases in general with higher load impedance. Furthermore, even with low current charging, the higher limit is around 0.37 A to 0.5 A, and we anticipate smaller current than this from our rectifier design, therefore SDP is better charging configuration for our applications.

### Current requirements for smartphone charging

Measurement so far reveals that power management and rectifier module is adaptable to low input resistance loads as shown in the Fig. 5. Furthermore, we notice from Fig. 6 that the standard downstream port impedance of the phone is a small resistance. We further validate the current requirement for charging of the phone. An experiment was conducted with fixed voltage and varying current values to determine the minimum required current for charging. The experiment showed that minimum voltage and current required for charging the phone are approximately 4.2 V and 62 mA respectively. Higher current levels can provide increasingly faster charging. The results are shown in Table 1.

**Table 1:** A typical smartphone’s current and voltage requirements for charging.

Voltage (V)	Current (mA)	Charging status
5	2.09	Blinking (charge decreases)
4.254	39	Stable (charge decreases)
4.262	62	Stable (battery life slowly increases)
4.279	103	Stable (battery life slowly increases)
4.306	167	Stable (battery life slowly increases)
5	939	Stable (battery life increases rapidly)
6.179	3.761	Blinking (charge decreases)

### Wireless power demonstrations using a single transmitter

Having designed the transmitter antenna array, the receiver, rectifier plus power management module, and the USB charging interfaces, we now conduct experiments for the wireless powering of the mobile device via a single transmitter source. At the receiver, we configure the system in the following manner. One patch antenna element is connected to one rectifier and power management module. In order to achieve the required current, we used several such rectenna modules. The DC converted output is then connected in parallel. This configuration allows improved power collection, since the power collection is done at the DC level which avoids the effect of phase variation in the received RF signal at different antennas.

To achieve the required current and provide the wireless charging, we consider the cases of 5 and 10 rectenna elements (Fig. 2) at the receiver. As shown in Table 2, for these cases the distance is varied between 1 foot to 4 feet and power is varied as 25 dBm, 30 dBm and 35 dBm. In general, we observe a trend of increase in duty cycle with increased number of elements. Nonetheless, 2 exception cases are noted, where duty cycle decreases upon an increase in the number of elements in the receiver array. These cases are associated to receiver power and location of (1) 25 dBm power at 3 feet, and (2) 35 dBm at 3 feet. We note that in the radiative near zone, the beam is not well formed, and it has a lateral distribution of power. Sufficient exposure of all elements under proposed variations of the experiment is not maintained for all cases. This is the expected cause of these anomalies. Note that the transmitted power from the antenna is 6 dB smaller than this supplied power, due to loss in the power divider network. In the case when the power is not sufficient to supply 5 V output, the power management circuit is designed to store the charges until voltage exceeds 5.3 V (turn on condition) and then supply is on until voltage drops below 4.2 V (turn off condition). Therefore, we observe a pulsed output, and the duty cycle is observed to increase with an increasing available input RF power. Notably, the phone’s charging was observed when the duty cycle was at least 25%. The duty cycle increases while the number of receiver antenna was increased to 10 from 5, which provided a higher duty cycle. Increased on to off ratio of the current is anticipated to increase the charging rate of the battery.

Comparing the results for the 5-element receiver with the 10-element receiver, on an average, the duty cycle improves due to larger power collection. Furthermore, as expected, the duty cycle and charging capability of the receiver has improved when the transmitted power is 35 dBm. We also note that in terms of distance variation, duty cycle and charging improve for 3 feet and beyond. This is because of the low efficiency charging in the near zone of the transmitter array, as is also clear from the power versus distance plot shown in Fig. 4. This is because of the lack of a coherent radiative power and a well-defined beam in the vicinity of the array. That is, the transmitter is most effective for receivers placed at distance of 3 feet or longer.

**Table 2:** Recording the duty cycle for varying wireless charging for the cases with varying distances between the Tx and Rx, and with varying input RF power using 5 Tx antennas (first 3 rows for Input power) and 10 Tx antennas (last 3 rows for Input power). The output voltage pulses were in the range of 4.2 to 5.5 V for all cases.

Input power (dBm)	Distance (ft.)	Duty cycle (%)	Charging status
25	1	10	No
	2	19	No
	3	45	Yes
	4	24	No
30	1	10	No
	2	26	Yes
	3	50	Yes
	4	40	Yes
35	1	10	Yes
	2	34	Yes
	3	60	Yes
	4	60	Yes
25	1	19	No
	2	20	No
	3	38.7	Yes
	4	44	Yes
30	1	19.2	No
	2	36	Yes
	3	54	Yes
	4	55.6	Yes
35	1	32	Yes
	2	44	Yes
	3	51	Yes
	4	65.5	Yes

**Figure 7:**
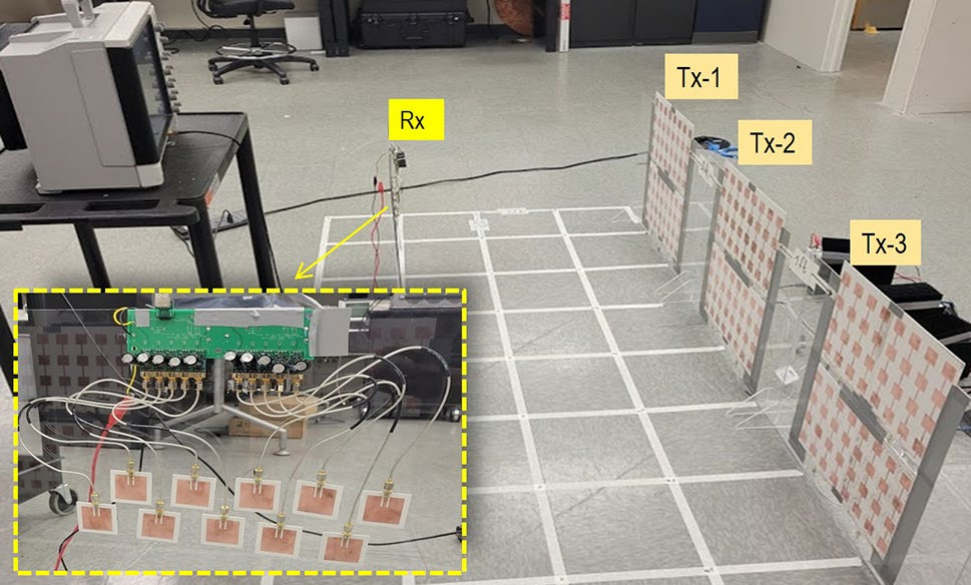
Experimental set-up to demonstrate the charging of the phone using the developed set-up.

**Figure 8:**
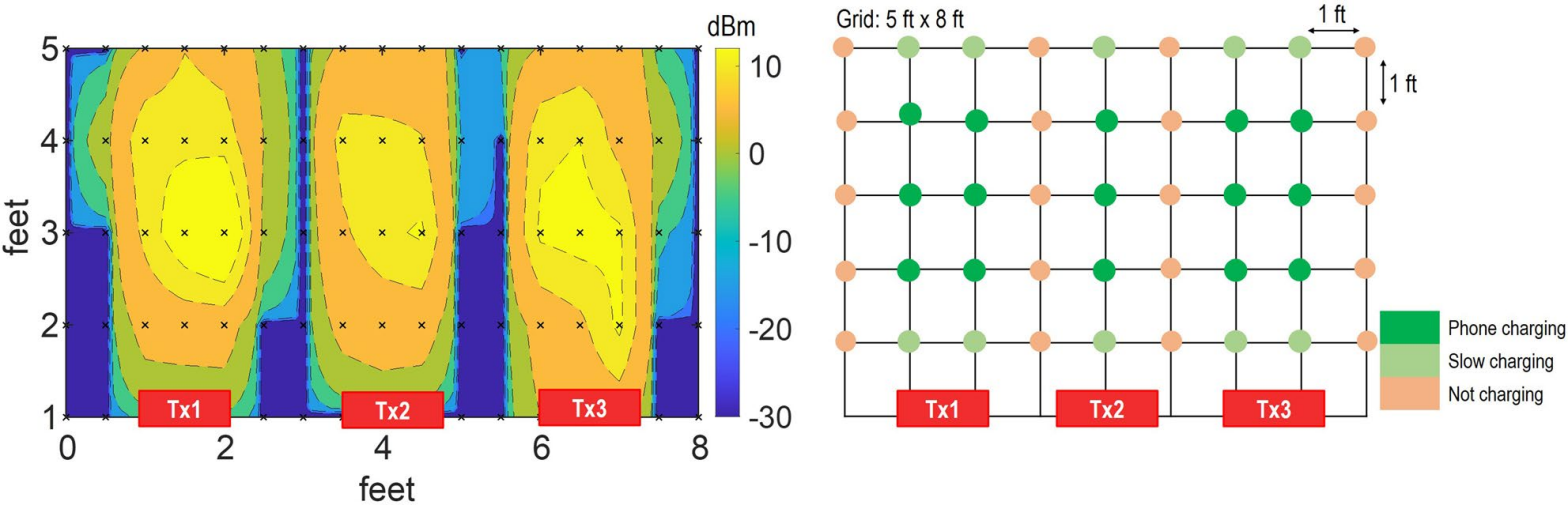
Wireless power grid with distribution of power. Left: power measured at 1 k$\Omega$ load. The cross points represent the measurement points and 2D plot is interpolated. Right: Charging ability of the power grid for a Samsung Galaxy phone. Plot generated using Matlab R2019b available at https://mathworks.com. Grid drawing is created using MS-PowerPoint 2016.

**Figure 9:**
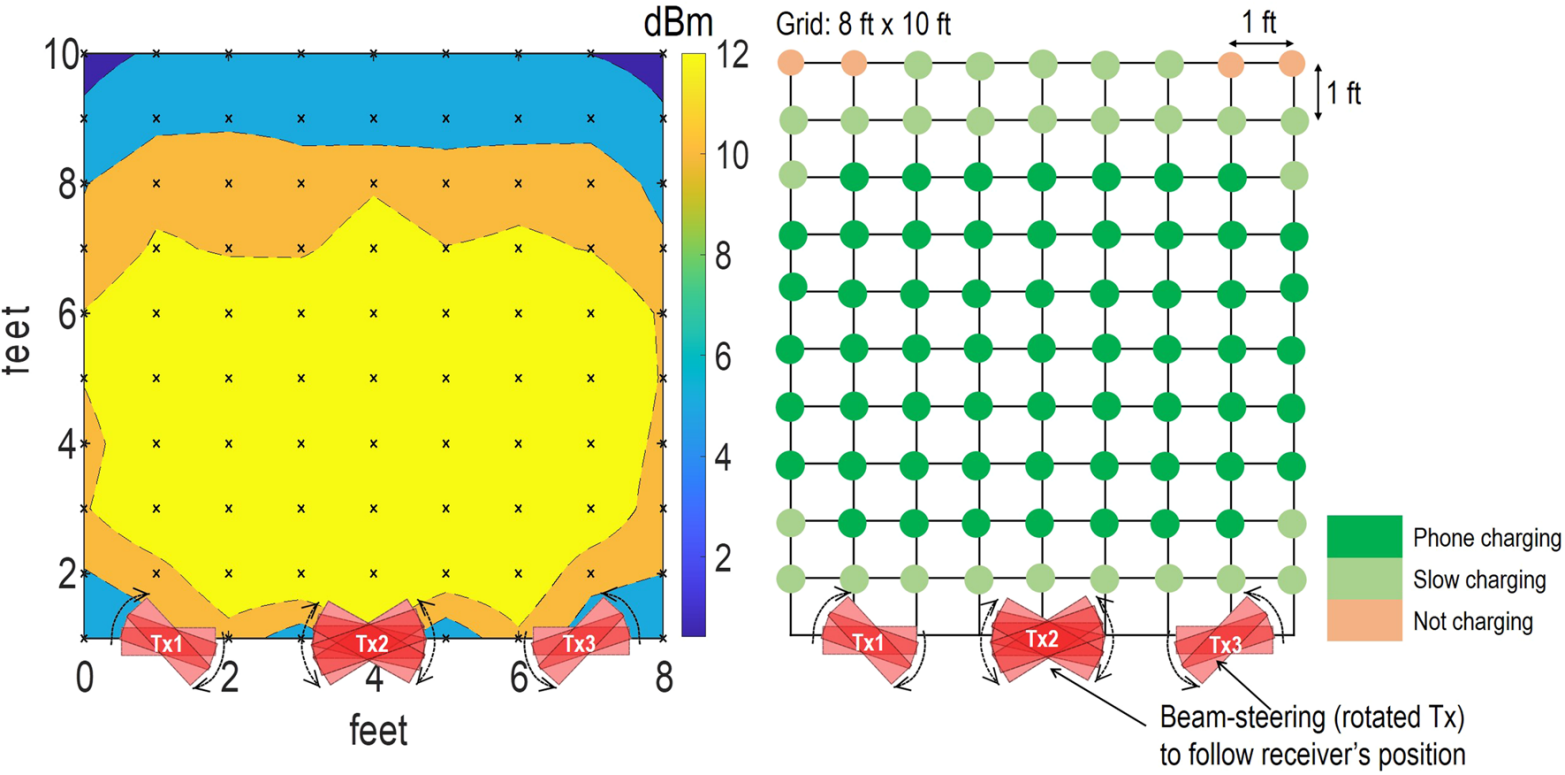
Power grid’s power distribution with transmitters having beam-steering capabilities. Plot generated using Matlab R2019b available at https://mathworks.com. Grid drawing is created using MS-PowerPoint 2016.

**Figure 10:**
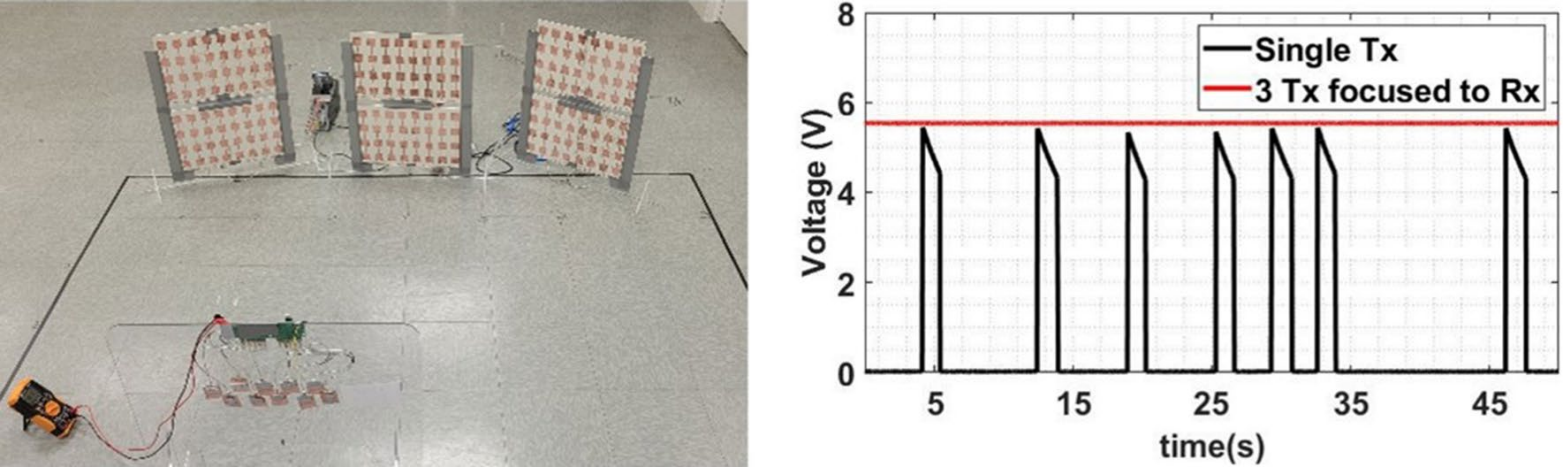
Duty cycle comparison from the power management unit for single Tx versus beam-steered multiple Tx scenarios. Plot generated using Matlab R2019b available at https://mathworks.com.

## Wireless power transfer grid demonstrations

Next, we show the use of many source panels to create the wireless power transmission grid and cover an area of 5 feet by 9 feet. For this demonstration, we consider three transmitters positioned in a line and with 1 foot edge to edge gap, shown in Figs. 7 and 8. In the meantime, the target receiver phone was moved in the grid. The three transmitter antennas were fixed at the edge of grid facing the grid with the signal generator and amplifier connected to the transmitters; the receiver system was placed at each point in the grid while facing the transmitter. The receiver was first connected to Keithley MSOS254A oscilloscope to measure the output power. Later the receiver system was connected to a smartphone using the USB 2.0 interface to show the charging capability of the grid.

1 W power was radiated by a transmitter using the signal generator with output of 0 dBm, followed by +30 dB amplification using a power amplifier (ZHL-42+ gain block). For the power measurements, 1 k$\Omega$ load was connected to the output of receiver and voltage waveform was read. The 2D representation of the power available in the grid is shown in Fig. 8 (left). Overall, the system allows collection of up to 10 dBm power and for distances up to 3 to 4 feet from the center of each transmitter. The power level decreases as we move closer to the transmitters, because the receiver is approaching the near field, where the beam is not well-formed. Similarly, the power level decreases when we move more than 5 feet away from the transmitters because of the propagation loss.

Charging capability of the system is shown by connecting the receiver to a Samsung galaxy S21 smartphone and then moving it across the grid. The results are shown in Fig. 8 (right). The phone charged at the distance of 2 feet to 4 feet in front of the each of the transmitter. The charging process slowed down while moving the receiver closer to or farther away from the transmitter. Similarly, we observe the null-zones, which lie in between the transmitters, where phone charging was not observed. The system demonstrates the charging ability, but we notice null zones in the grid. We account for these null-zones by proposing the use of beam-steering such that the beam can follow the receiver target.

The beam-steered transmitter grid is evaluated by mechanical rotation of the transmitter arrays, such that all three transmitters are pointed towards the receiver for all positions of the receiver in the grid. The results from this measurement are shown in Fig. 9. In our concept of Wireless Grid LAN, the beam steering of the antenna not only fills the gaps in the measurements, but it also provides added power due to cumulative addition of incident power from all three transmitters. We observe a minimal effect of power decrease with potential phased interference from the three sources. Figure. 9 (left) shows that we have continuous supply of power in this case, and Fig. 9 (right) shows that the phone’s charging is retained across the grid for all positions of the receiver. Furthermore, we noticed that power delivery is improved in the near field region of the transmitters (compare Figs. 8 and 9). This is because near field zone of transmitter-one lies in the far field zone of transmitter-two and three. Since, antennas have well-formed beam in the far-field, use of beam-steering allows better power delivery closer to the transmitter. In our experiment here, we measured the cumulative addition of power from three transmitters, but another tracking and handover modality is possible. That is, the experiments point to the utility of tracking the receiver and providing handover ability to a nearby Tx, as the device moves out of the range of the first Tx. Such envisioned systems, based on this experiment, will have significant practical applications.

Quantitatively the improvement in beam-steered Tx system is assessed by measuring the duty cycle of the received signal for the cases when the receiver obtained power from a single transmitter (Fig. 7) and then from three transmitters (Fig. 10). We notice an increased duty cycle as shown in Table 3. For only single transmitter, we don’t see 100 % duty cycle in any position. But, when all three transmitters are steered towards the receiver, the duty cycle is continuously retained at 100 % for spacing between transmitter and receiver up to 7 feet. After 7 feet, the duty cycle is less than 100 % and we start to see the waveform, although the duty cycle is still higher than the cases with single transmitter. Figure 10 (right) shows the voltage waveform read using oscilloscope with 2 feet spacing between transmitter and receiver for both cases. As seen, the power management circuit is continuously on for charging of the mobile device in question. We note that for both systems, i.e., systems with and without beam-steering, optimum performance is achieved when the distance the Tx-Rx distance is between 3 and 5 feet. Furthermore, the power available near the antenna (less than 3 feet) is higher with beam steering as compared to the one without beam steering. The power level decreases while moving close to transmitters because the receiver is approaching the near field and the beam is not well formed. But for the system with beam steering, while the receiver is in near field of a transmitter directly in front of one, it is in radiating near field of the adjacent one, hence, more power is available.

**Table 3:** Duty cycle and voltage of DC signal at the output of receiver while receiver is placed in front of single transmitter (2nd row) and while all three transmitter is focused on the receiver (3rd row).

Distance Tx-Rx (feet)	Duty cycle in front of Tx /(Harnessed voltage)	Duty cycle with 3 Tx focused to Rx /(Harnessed voltage)
3	34.36 % (5.44–4.16 V)	100 % (5.12)
4	45 % (5.44–4.16 V)	100 % (5.05 V)
5	41 % (5.44–4.16 V)	100 % (5 V)
6	18.2 % (5.44–4.16 V)	100 % (4.9 V)
7	11 % (5.44–4.16 V)	91.5 % (5.44–4.16 V)
8	9 % (5.44–4.16 V)	74.5 % (5.44–4.16 V)

## Conclusions

The presented hardware development and related studies have demonstrated a wireless power grid, which can seamlessly charge mobile devices. The same concept can be used to charge sensors, IoT and wearable devices. The system is of great advantage from an ergonomic usability point of view, as it provides a continuous charge without having to plug in the devices. Current report demonstrated that three transmitters each with 1 W output, connected to an antenna panels of the size 48 cm by 60 cm, have ability to cover wireless charging needs in a grid of area 8 ft by 10 ft. The report demonstrated a prototype of power grid concept by an effective integration of antenna, rectifiers, power management circuit, and USB 2.0 interface for a practical power charging set-up. Notably, commercially available smartphone was charged continuously in the grid using the proposed wireless power grid system. In future work, the current generation of mechanically beam-steered antennas should translate into an electronic beam steering or beam-switching solution. Furthermore, the size of the receiver array can be reduced using a compact board design for integration into a practical use. The number of receiver elements can further be reduced by using a more efficient RF-DC converter and power management circuit, as opposed to COTS circuits used in this demonstration. These developments are imminent as the current hardware prototype demonstrates the feasibility and serves to open way into realistic future wireless power grid.
